# Personalized medicine in patients with Osteogenesis imperfecta

**DOI:** 10.1186/2194-7791-1-S1-A10

**Published:** 2014-09-11

**Authors:** Oliver Semler, Heike Hoyer-Kuhn, Joerg Doetsch, Eckhard Schoenau

**Affiliations:** Children´s Hospital, University Cologne, Cologne, Germany

## Background

Due to an expanding knowledge about the molecular diversity the disease Osteogenesis imperfecta (OI), formerly known to be a disorder of the collagen synthesis, changed to a group of disorders with a fragile skeleton as common symptom. Patients with OI type VI are clinically characterized by an increased amount of osteoid and a poor response to bisphosphonate treatment.

## Patients

In two boys with signs of OI we identified the disease causing mutation of OI VI [1]. Mutations in SERPINF1 lead to the absence of pigment epithelium derived factor (PEDF), which is a key factor for the regulation of osteoclastogenesis. Without PEDF the differentiation of pre-osteoclasts is increased and more bone will be resorbed.

## Consequence

Due to the new understanding of the pathophysiology of OI VI we decided to target the osteoclasts directly using the Rankl-antibody Denosumab. This was the first use of Denosumab in children.

## Results

With this change of therapy we were able to reduce bone resorption to the normal range [2].

After two years of treatment both patients show an increase of bone mineral density of the lumbar spine with a z-score from – 2.4 to -2.0 and -4.3 to 2.5 respectively and an increase of projected vertebral area L2 – L4 [mm²] from 1670 to 2152 and from 1424 to 1528. No severe side effects were seen and the drop of serum calcium after the subcutaneous injection could be treated by oral calcium supplementation.

## Conclusion

Clinical observation and molecular analysis lead to a new understanding of OI VI and to a more specific therapeutic approach. The new therapy resulted in a better therapeutic effect and is a striking example of a fast transfer from basic research to therapeutic use.
